# Lung Health in Children in Sub-Saharan Africa: Addressing the Need for Cleaner Air

**DOI:** 10.3390/ijerph17176178

**Published:** 2020-08-26

**Authors:** Refiloe Masekela, Aneesa Vanker

**Affiliations:** 1 Department of Paediatrics and Child Health, Nelson R Mandela School of Clinical Medicine, College of Health Sciences, University of KwaZulu-Natal, Durban 4013, South Africa; 2 Department of Paediatrics and Child Health, Red Cross War Memorial Children’s Hospital and MRC Unit on Child and Adolescent Health, University of Cape Town, Cape Town 7700, South Africa; Aneesa.vanker@uct.ac.za

**Keywords:** pollution, indoor, outdoor, lung health, respiratory tract infection

## Abstract

Air pollution is increasingly recognized as a global health emergency with its impacts being wide ranging, more so for low- and middle-income countries where both indoor and outdoor pollution levels are high. In Africa, more than 80% of children live in households which use unclean sources of energy. The effects of both indoor and outdoor pollution on lung health on children who are the most vulnerable to their effects range from acute lower respiratory tract infections to long-term chronic health effects. We reviewed the literature on the effects of air pollution in children in Sub-Saharan Africa from prenatal exposure, infancy and school-going children. Data from Sub-Saharan Africa on quantification of exposures both indoor and outdoor mainly utilizes modelling or self-reporting. Exposures to biomass not only increases the risk of acute respiratory tract infections in young children but also increases the risk of carriage of pathogenic bacteria in the upper respiratory tract. Although there is limited evidence of association between asthma and pollution in African children, airway hyper-responsiveness and lower lung function has been demonstrated in children with higher risk of exposure. Interventions at a policy level to both quantify the exposure levels at a population level are urgently needed to address the possible interventions to limit exposure and improve lung health in children in Sub-Saharan Africa.

## 1. Introduction

Air pollution is increasingly recognised as a global health emergency, which disproportionately affects low and middle-income countries (LMICs) with nearly 92% of deaths occurring in these countries [1]. The impact of air pollution on child health is significant, starting in utero, through infancy, childhood and progressing to lifelong health effects [2,3].

Air pollution comprises both outdoor and indoor sources, while ubiquitously affecting all population groups globally. In areas with higher exposure levels, the health impacts are more significant [4]. Ambient or outdoor air pollution arises from both unnatural and natural sources [1,5] including industrial processes, fossil fuel combustion [6], agricultural practice [7], traffic [8] and wildfires [9], dust storms [10] and volcanic eruptions [11,12,13]. Indoor or household air pollution is the result of the use of alternate and polluting fuels as a source of household energy for cooking and heating [14]. These include solid, biomass and fossil fuels [15,16] as well as kerosene (paraffin) [17,18]. Incomplete combustion often combined with inadequate ventilation of these polluting substances results in a large number of chemical compounds such as particulate matter (PM), sulphur dioxide, nitrogen dioxide, carbon monoxide (CO) and volatile organic compounds that are then inhaled [19,20]. It is usually a combination of both outdoor and indoor sources that impact on child health. While there has been a universal shift towards electrification and access to cleaner fuels, this is closely linked to socioeconomics, with many African countries still relying on polluting fuels for household activities [14,18]. Further, outdoor air pollution is often poorly regulated in African countries, with no formal measurements done in most countries and poor compliance from industries [21,22].

The burden of respiratory diseases in African children is high. Despite sustained efforts to address this, lower respiratory tract infections (LRTI) remain the leading cause of childhood mortality, particularly in this region [23]. However, the burden of non-communicable diseases is also increasing, with asthma being the most common chronic respiratory disease in children [24,25,26,27,28,29,30,31,32,33,34,35].

Understanding the impact of air pollution on lung health in children is paramount, not just to reduce the effects on acute respiratory conditions but also to address the long-term sequelae of these exposures. This paper aims to review the literature on air pollution and its impact on lung health in African children.

## 2. Methodology

**Figure 1:**
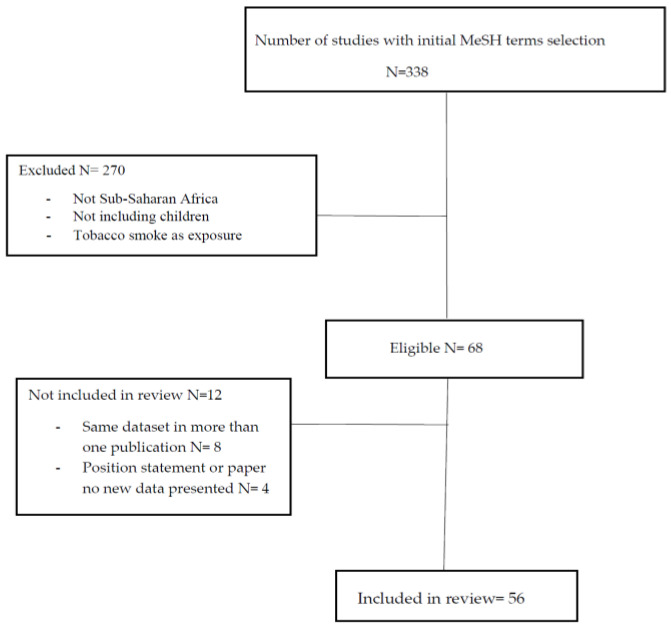
Studies reviewed and included on air pollution in Sub-Saharan Africa.

A search on Pubmed, Google Scholar and African Index Medicus was performed over a period of 20 years from January 2000 to December 2020. The search terms included the following MesH (Medical Subject Headings) terms or a combination of these: infant, children, adolescent, air pollution, indoor, outdoor, maternal, exposure, lung health, acute respiratory tract infections, asthma, tuberculosis, chronic lung disease and bronchiectasis and Africa/and or Sub-Saharan Africa. Only publications in English with full text were included in the search. Abstracts generated were reviewed by the co-authors and 338 publications relevant to the topic were included in the final review. Articles were reviewed if they contained data on either indoor or outdoor pollution as an exposure and included either acute or chronic respiratory disease as an outcome. Only studies conducted in Sub-Saharan Africa were reviewed. Papers were excluded if they included tobacco smoke exposure or were from the North Africa region. After exclusion, 68 papers were reviewed of which data on 56 papers were included, Table 1 and Table 2 and Figure 1.

## 3. Epidemiology

Air pollution is referred to as Africa’s “silent killer” with an estimated 600,000 deaths annually [22]. The World Health Organization (WHO) reports that 100% of children under 5-year of age are exposed to levels of fine particulate matter above WHO acceptable ambient standards and 83% of African children live in homes that rely on the use of polluting fuels for household energy [18]. Indoor air pollution rates are high with African countries reporting high levels of biomass fuel use ranging from 5% to 95% depending on the region.

One of the major limitations is the paucity of measured exposure data from Africa, with many studies relying on reported exposures or modelling data [36,37]. However, the limited published data confirm high exposure levels. A study from Uganda, located in east Africa, measured ambient air pollution in two Ugandan cities, including the capital Kampala, and found the mean particulate matter (PM_2.5_) concentration was 5.3 times the World Health Organization (WHO) cut-off limits [38].

This is further shown in what appears to be the only systematic review of air pollution in Sub-Saharan Africa, where measured ambient air pollution levels were 10–20 times higher than acceptable WHO standards. The limitation of this review is that most of the studies included were from South Africa which compared to other Sub-Saharan African countries had lower indoor pollution levels. Further, most Sub-Saharan African countries contributed no data, highlighting the lack of African data [39].

In terms of household exposure to indoor air pollution, a large cross-sectional study of more than 16,000 households in Malawi found that the use of biomass fuels predominated with 81.5% of households using charcoal for energy and only 3.9% of homes using electricity exclusively [40]. The authors concluded that this was similar to many peri-urban Sub-Saharan areas and disproportionately affected people of lower socioeconomic status.

## 4. Pathophysiology

The impact of air pollution on child lung health begins in utero with the developing foetus. There are a number of mechanisms by which air pollution exposure during pregnancy affects long-term respiratory health, including the impairment of organogenesis and organ development, indirectly affecting lung development by causing premature birth, lower birth weight and disturbed development of the immune system [41]. Air pollution in pregnancy also impacts on lung function, recurrent respiratory tract infections and the development of asthma. Whilst the mechanism for these is not fully understood, it is thought to be through a complex interplay of environmental and epigenetic factors, involving both the direct effects of air pollution on the placenta resulting in systemic inflammation in the mother. Further, nanoparticles from air pollution may cause both developmental and epigenetic changes [41].

There are few African studies that assess the effects of air pollution exposure in early life on lung health. The Drakenstein Child Health Study, a South African birth cohort study, assessed the impact of prenatal exposures on infant lung function at 6 weeks of age and found that antenatal exposure to benzene altered lung function (lower time to peak tidal expiratory flow over total expiratory time ratios; 3.0% (95% CI −5.2% to −0.7%, *p* = 0.01)) [42]. From the same birth cohort, antenatal exposures to particulate matter and toluene (a volatile organic compound) were associated with both the development and severity of lower respiratory tract infections in infancy [43].

Children’s susceptibility to air pollution is increased due to a number of factors including, vulnerability of the developing airways, an immature immune system, increased ventilation and greater proportion of time spent indoors compared to adults [44]. Air pollution affects respiratory defence mechanisms, causes epithelial inflammation allowing for organisms to breach the epithelial barrier more easily and ultrafine particles can rapidly pass into the systemic circulation inducing a systemic response.

In terms of gene–environment interactions, the genome wide association studies (GWAS) have identified a number of alleles associated with asthma risk. The applicability of this is, however, more complex [45]. Air pollution, in itself, impacted on airway mechanics, and reducing exposures to particulate matter (PM_2.5_) had significant effects on airway resistance and inflammation [46]. New evidence from Africa found that both antenatal and postnatal air pollution (PM_10_) exposure reduced lung function at 6 weeks and 1 year of age. In these infants with a genetic predisposition to asthma, there was an increased susceptibility to the adverse effects of prenatal exposure to indoor air pollution, demonstrating the importance of gene–environment interactions for both infant lung function and longitudinal lung health [47].

## 5. Exposure Assessments

Quantitative assessment of indoor air pollution can be made through personal measures of known by-products of combustion or from using biomarkers of pollutant exposures. Both these methods can be expensive or invasive, with equipment often unsuitable for use in young children [48,49]. A study in the rural areas of Uganda and Ethiopia measured particulate matter (PM_2.5_) and CO across age groups and gender. The study found that women and girls had much higher household pollution exposure levels compared to men and boys, a reflection of involvement in household cooking activities and time spent indoors [50]. Another peri-urban study in South African assessed indoor air pollution by measuring the by-products of combustion in over 600 homes, finding that despite access to electricity in the majority of homes, fossil fuels were still used for cooking and heating in up to 30% of homes [51]. Further, the median benzene (a volatile organic compound) levels were significantly above acceptable ambient standards and this together with increased CO and nitrogen dioxide levels were associated with fossil fuel and kerosene (paraffin) use [51].

A study from The Gambia assessed the correlation between direct measurement versus indirect modelling of children exposed to particulate matter from biomass burning, found that although children were exposed to very high PM_2.5_ levels, there was poor correlation (correlation coefficient 0.01) between direct and indirect measures [52], again highlighting that direct measures of exposure are most accurate and necessary to correlate with health risks and outcomes.

Biomarkers of exposure may be difficult in children, especially if they are invasive or require cooperation. As part of the Cookstove and Pneumonia Study (CAPS), a large community-level cluster randomised control trial in Malawi, measured levels of CO in homes and carboxyhaemaglobin (COHgB) in children under 5 years were conducted at baseline and after a cookstove intervention. COHgB levels were high (5.8% (3.3; 0–20.3)) at levels associated with adverse health outcomes; however, there was a poor correlation between CO and COHgB level (Spearman’s = 0.09, *p* < 0.001), suggesting that CO exposure was cumulative and from a number of sources, not just household air pollution [53].

## 6. Lung Health Impacts

### 6.1. Acute

Indoor and outdoor air pollution have significant health effects with reported increases in cough, persistent cough, wheezy episodes and acute respiratory tract infections (ARIs) particularly in young children under the age of 5 years [54,55,56,57,58], Table 1. In the Zimbabwean Demographic and Health Survey data on children under the age of 5, exposure to high levels of biomass in over 3550 children was associated with a more than 2-fold risk of ARI, this risk was highest in the 6–11-month age group [58]. In another study in Bamenda Northwest Cameroon in adults and children (57% infants), exposure to indoor air pollution was associated with a 3.6-fold risk of developing an ARI [54]. Exposure to indoor air pollution was also high in women and increased their risk for ARI. Another study in Tanzania found that women and children under 5 years of age were found to have a more than 5-fold risk of ARI when exposed to indoor pollution from biomass [55].

It is known that biomass exposure doubles the risk of pneumonia in children under the age of 5 years [61]. Exposure to biomass may also be related to an increased carriage of pathogenic organisms in the respiratory tract. A birth cohort study found exposure of young infants to particulate matter in the first year of life was associated with increased carriage of *Moraxella catarrhalis* and *Haemophilus influenzae* [72].

In the context of outdoor air pollution, children are exposed to a number of sources of pollution including burning of rubbish, agricultural field burning, traffic-related pollution and outdoor cooking [68]. A number of studies have shown that living next to a road with high traffic density, more so truck-related traffic, is associated with increased risk of dry cough and reported wheeze [60,65,66]. A study of South African children in a highly industrialized area under 26 months of age found an adjusted odds ratio of 3.88 (95% Confidence Interval 2.29–6.57) of dry cough if living next to a road with high truck density [65]. A similar study in the same population conducted in school age children 13–14 years of age found an association between high truck traffic and current wheezing [60].

### 6.2. Long-Term Lung Health Effects and Asthma

It still remains unclear whether there is a linear relationship between exposure to biomass fuels and lung function in children. Intervention studies with control groups in Guatemala have shown a lower but not statistically significant rate of lung growth in exposed versus intervention children in the first 6 months of life [66]. A study in Malawi on school-going children in the Cooking and Pneumonia sub-study found lower FVC z-scores in children with higher exposure households although this was a small difference with borderline significance (0.22 z-score), but not for any other lung parameters [71]. In a recent analysis of the Drakenstein Child Health study, infants with a genetic predisposition for asthma were more susceptible to the adverse effects of prenatal PM_10_, and this interaction also depended on the infants’ ancestry with infants of black African ancestry being more vulnerable [47].

There are conflicting data regarding the association between biomass exposure and asthma. A study in Nigerian school children found no association between exposure to woodsmoke and asthma prevalence and lung function [62]. With regard to airway mechanics, a small study on asthmatic children found that changes in exposure to PM_2.5_ was associated with increases in airway resistance but not other spirometric variables including forced expiratory volume in the first second (FEV1), forced vital capacity (FVC), peak expiratory flow (PEF), forced expiratory flow at 25–75% of the FVC (FEF25-75), and the ratio between FEV1 and FVC (FEV1/FVC) [47]. Exposure to kerosene as an indoor pollutant was found in one study to be associated with an increase in FeNO, a marker of airway inflammation in children [18]. With regard to outdoor exposures a study in Durban, South Africa found that increasing and fluctuating levels in NO and NO_2_ levels were associated with significant declines in FEV_1_ in school-going children, and this effect was exaggerated in those children with airway hyper-responsiveness or persistent asthma [70]. Interestingly, a recently published study from Cape Town, South Africa, using land-regression models, found that even with nitrogen dioxide (NO_2_) levels below acceptable ambient standards, there was an association with asthma associated outcomes at twelve months, independent of particulate matter PM_2.5_ levels [73].

## 7. Impact of Interventions to Reduce Air Pollution

Addressing reduction in air pollution exposure is not simple. It is increasingly evident that access to clean air for all requires a multi-pronged approach [18]. In Africa, where cooking with biomass and other unclean fuels is common, a number of studies has explored cookstove interventions to try and reduce household air pollution [14]. This involves providing households with cooking stoves that use less-polluting fuels and combust more efficiently. However, a complex combination of factors has precluded the widespread uptake and benefit of this intervention alone, including economic status, lack of commitment, cultural views and concern with safety and security [14]. The health benefits of this intervention alone have also been disappointing. The large Cookstove and Pneumonia Study (CAPS) in Malawi, used a community-level open cluster randomised controlled trial to compare the effects of a cleaner burning biomass-fuelled cookstove intervention to continuation of open fire cooking on pneumonia in children and included 10,750 children from over 8000 households. The study found no evidence that an intervention comprising cleaner burning biomass-fuelled cookstoves reduced the risk of pneumonia in young children [67]. Similar results have been noted from other African cookstove intervention studies from Kenya [63] and Rwanda [63]. In both these studies, the most likely factor was the uptake and sustained use of the cookstove intervention. In the Rwandan study, the frequency of the intervention stove use decreased from 72.9% to 52.5%, with an inverse increase in the traditional stove use during the study period. As a result, there was also no significant reduction on measured particulate matter (PM_2.5_) levels [69]. Similarly, in the Kenyan study despite the relatively low cost of the intervention stove, economic constraints still played a role in the widespread uptake, with less than 20% of homes using it. Further, many homes continued to use traditional stoves or a combination of traditional and the intervention stove, which may also have played a role in there being no significant decrease in measured PM_2.5_ levels and while children from homes using the intervention stove had fewer reported respiratory symptoms, this was not significant [73].

A critical component to reducing air pollution exposure includes behaviour changes which relate to cooking outdoors, improving ventilation in the cooking area and keeping children away from smoky environments as potential strategies that can be implemented without too much additional expense. A review of the impact of behaviour change strategies on indoor air pollution and childhood respiratory health from LMIC, found that behavioural change had the potential to reduce indoor air pollution; however, of the studies available, many of the methodologies were weak. The review concluded that more robust studies were required to assess this using potentially available behaviour strategies [64].

## 8. Discussion

In this review of air pollution and lung health in African children, the authors found that, despite limited data, the impact of both indoor and outdoor air pollution on acute and chronic childhood respiratory conditions was significant. While large-scale measured exposure data are few, studies which assessed air pollution levels showed that ambient air pollution levels were up to 20 times higher than WHO accepted ambient standards.

The increased susceptibility of children is also evident with air pollution, impacting on lung health from in utero and early life with longitudinal consequences, highlighting the urgent need to address all sources of air pollution. As in many African countries, there are often “communities in transition” with changing geographic and socio-economic status, a result of migration of communities from rural to peri-urban settings and from economic upliftment. Although households may gain cleaner and more efficient fuels, a combination of both polluting and non-polluting fuels are often used known as fuel stacking [74]. The ultimate intervention is to ensure universal access to “affordable, reliable, sustainable and modern energy for all” by 2030, in keeping with the seventh sustainable development goals [18]. Strict and enforced government regulations that enforce ambient air standard levels by ensuring industry compliance are also necessary to reduce the health impacts of air pollution [4,21].

The impact of these exposures on genetic, epigenetic and immunological changes requires further investigation in an African setting where we face a “colliding epidemic” of both infectious diseases and environmental exposures [59]. Studies from high income countries have reported the impact of early-life exposures on the genetic programming that control life-long lung development, aging and susceptibility to obstructive lung diseases [24,44].

## 9. Conclusions

Data particularly on exposure assessments are desperately needed both for indoor and outdoor exposures in Sub-Saharan Africa. There is a need to greater advocacy for cleaner fuel sources in Sub-Saharan Africa and for countries to utilize behaviour change to reduce early life exposures, particularly to indoor pollution in young children to mitigate against the burden of lower respiratory tract infections and future lung health impacts. A more stringent regulatory environment is required with regards to industry and vehicle emissions to mitigate outdoor pollution effects.

## Figures and Tables

**Table 1:** Selected papers for inclusion and publication year.

Publication Year	No Publications	Author	Summary
2002	1	Ezzati M [49]	Solid fuels and health impacts.
2003	1	Mishra V [58]	BMF and ARIs in presechool children.
2007	1	Kilabuko JH [55]	Air quality and ARSs in chldren.
2010	1	Van Zyl-Smit R [59]	TB, smoking, HIV and COPD.
2011	2	Mustapha BA [60]	Air pollution and respiratory illnesses.
		Po JY [61]	Respiratory diseases and BMF exposure.
2012	1	Dionisio KL [52]	Exposure of children to PM in household.
2013	4	Adeloye D [24]	Asthma prevalence in Africa.
		Gall ET [48]	Air pollution in developing countries.
		Thacher JD [62]	BMF and asthma risk.
		Foote [63]	Cookstoves and impact on lung health
2014	6	Barnes BR [64]	Behavioural factors and air pollution.
		Gordon SB [14]	Respiratory risk for HAP.
		Piddock KC [40]	BMF in Malawi.
		Sanbata H [57]	BMF and ARI in children under 5 years.
		Shirinde J [65]	Wheeze and air pollution in children.
		WHO [19]	Air pollution and burden of disease
2015	4	Kirenga BJ [38]	Air quality in two Ugandan cities.
		Sly PD [3]	Early origins of COPD.
		Vanker A [51]	Home environment and air pollution exposure.
		Voynow JA [44]	Air pollution and the developing lung.
2016	3	Heinzerling AP [66]	Lung function and wood exposure.
		Goldizen FC [25]	Respiratory effect of air pollution on children.
		Ober C [45]	Asthma and GWAS studies.
2017	5	Gray D [42]	Early lung function determinants.
		Korten I [41]	Air pollution in pregnancy and lung development.
		Mortimer K [67]	Cleaner cookstoves and impact on pneumonia.
		Vanker A [43]	Early life exposures and ARI and wheezing.
		WHO [18]	Inheriting a sustainable world.
2018	9	Admasie A [56]	Under 5 years and air pollution.
		Arku RE [36]	HAP exposure and rural-urban differences.
		Havens D [53]	Carbon monoxide exposure in children under 5 years.
		Kim D [2]	Early origins of lung disease and air pollution.
		Landrigan [1]	Pollution and health.
		Okello G [50]	Women and girls increased pollution exposure.
		Olutola BG [68]	Cough and wheeze and outdoor air pollution.
		Shupler M [37]	Global estimates of HAP.
		WHO [19]	Air pollution and child health
	
2019	11	Atani M [21]	Air pollution and mortality.
		Katoto P [39]	Ambient air pollution and health in sSA.
		Kirby MA [69]	Cookstoves and ARI.
		Mentz G [70]	Effect modifiers of lung function and air pollution.
		McAllister DA [23]	Global, regional and national estimates of LRTI mortality.
		Myllyvirth L [21]	Air quality and health impacts.
		Nsoh M [54]	ARI and air pollution.
		Olaniyan T [18]	Air pollution and respiratory health of children.
		Rylance S [71]	Lung health and air pollution in children.
		Schraufnagel DE [4]	Health benefits of air pollution reduction.
		Vanker A [72]	Indoor air pollution and bacterial carriage.
2020	3	Hüls A. [47]	Genetic susceptibility to asthma and air pollution.
		He L. [46]	Perinatal air pollution exposure and respiratory mechanics in children.
		Olaniyan T [73]	PM exposure and NO on respiratory health of children.
	

Key: Tuberculosis (TB), Human immunodeficiency virus (HIV), Chronic obstructive pulmonary disease (COPD; LRTI: Lower respiratory tract infection; AR: Acute respiratory infection; sSA: sub-Saharan Africa; NO: nitric oxide; HAP: household air pollution; BMF: biomass fuels.

**Table 2:** Impact of air pollution on lung health.

Country	Indoor vs. Outdoor Source	Pollutant	Timing of Exposure	Age of Effect	Effect	Study
South Africa	Indoor	Benzene	Prenatal	6 weeks	Altered lung function (lower time to peak tidal expiratory flow over total expiratory time ratios; 3.0% (95% CI −5.2% to −0.7%, *p* = 0.01))	Determinants of early-life lung function in African infants [42].
Zimbabwe	Indoor	Biomass	Infancy	6–11 months	Increased risk of Acute Respiratory Infections (two-fold)	Indoor air pollution from biomass combustion and acute respiratory illness in preschool age children in Zimbabwe [58].
Cameroon	Indoor		Infants	Mean age 9 years	Increased risk of Acute Respiratory Infections (OR 3.62, 95% CI 1.45–4.90)	Acute respiratory infection related to air pollution in Bamenda, North West Region of Cameroon [54].
Tanzania	Indoor	Biomass PM_10_, NO_2_, and CO	Children under 5years and household	Under 5 years and exposed women cooks	ARI prevalence for cooks and children under age 5 making up the exposed group—54.7% (OR 5.5; 95% CI 3.6 to 8.5)	Air quality and acute respiratory illness in biomass fuel using homes in Bagamoyo, Tanzania [55].
South Africa	Indoor	Particulate matter (PM_10_)	Postnatal	Infants	Nasopharyngeal carriage of *H. influenzae* (aRR 1.68 (95% CI 1.10–2.57)) or *Moraxella catarrhalis* (aRR 1.42 (95% CI 1.03–1.97))	Indoor air pollution and tobacco smoke exposure: impact on nasopharyngeal bacterial carriage in mothers and infants in an African birth cohort study [72].
South Africa	Outdoor	TRAP	Children under 5 years	Aged 1 to 26 months	Children with trucks passing on streets frequently, dry cough more likely (aOR 3.88; 95% CI 2.29–6.57).	Factors associated with parent-reported wheeze and cough in children living in an industrial area of Gauteng, South Africa [68].
South Africa	Indoor and Outdoor	Gas heating and cooking TRAP	School going children	13–14 years	Gas used for residential heating —“wheeze ever” increased by 47% (OR 1.47 95% CI: 1.15–1.88). Trucks passing near homes—increased “wheeze ever” (OR 1.32 95% CI: 1.01–1.73), “current wheeze” (OR 1.61 95% CI: 1.15–2.24) and “current severe wheeze” (OR 2.22 95% CI: 1.28–3.77).	Association between wheeze and selected air pollution sources in an air pollution priority area in South Africa: a cross-sectional study [65].
Nigeria	Outdoor	TRAP	School going children	7–14 years	Traffic disturbance at home (noise or fumes) associated with wheeze (OR = 2.16; 95% CI 1.28–3.64); Night cough (OR 1.37; 95% CI 1.03–1.82) Phlegm (OR1.49; 95% CI 1.09–2.04)	Traffic air pollution and other risk factors for respiratory illness in schoolchildren in the Niger-delta region of Nigeria [60].
Malawi	Indoor	Carbon monoxide–household air pollution	School going children	6–8 years	Spirometric abnormalities (7.1% low forced vital capacity (FVC); 6.3% obstruction) were seen in 13.0% of children	Lung health and exposure to air pollution in Malawian children (CAPS): a cross-sectional study [71].
South Africa	Indoor	PM_10_	Prenatal and postnatal exposure	Infants 6 weeks and 1 year	Pre- and postnatal PM_10_ exposure reduced lung function at 6 weeks and 1 year and lower respiratory tract infection in the first year. Infants with asthma-related risk alleles more susceptible to PM_10_-associated reduced lung function.	Genetic susceptibility to asthma increases the vulnerability to indoor air pollution [47].
South Africa	Indoor	Kerosene (paraffin)	School going children	9–11 years	Kerosene use associated significant airway inflammation (FeNO > 35 ppb) (aOR: 2.31, 95% CI: 1.05–5.06).	Asthma-related outcomes associated with indoor air pollutants among schoolchildren from four informal settlements in two municipalities in the Western Cape Province of South Africa [17].
South Africa	Outdoor	NO_2_ and PM_2.5_	School going children	Grade 4 pupils	NO_2_ associated with increased risk of: new onset of ocular-nasal symptoms (aOR: 1.63, 95% CI: 1.01–2.60), wheezing (aOR: 3.57, 95% CI: 1.18–10.92), ≥2 asthma symptom score (aOR: 1.71, 95% CI: 1.02–2.86), and airway inflammation FeNO > 35 ppb (aOR: 3.10, 95% CI: 1.10–8.71), independent of PM_2.5_ exposures.	The association between ambient NO_2_ and PM_2.5_ with the respiratory health of school children residing in informal settlements: A prospective cohort study [73].

Key: acute respiratory infections—ARI; particulate matter 10 μg—PM_10_; nitrogen dioxide—NO_2_; carbon monoxide—CO; TRAP—traffic related air pollution; odds ratio—OR; 95% confidence interval—95% CI; aOR—adjusted odds ratio; FENO—exhaled nitric oxide.
